# A Reduced Complexity Acoustic-Based 3D DoA Estimation with Zero Cyclic Sum

**DOI:** 10.3390/s24072344

**Published:** 2024-04-07

**Authors:** Rigel Procópio Fernandes, José Antonio Apolinário, José Manoel de Seixas

**Affiliations:** 1 Program of Defense Engineering, Military Institute of Engineering (IME), Rio de Janeiro 22290-270, Brazil; apolin@ime.eb.br; 2 Department of Electrical Engineering, Military Institute of Engineering (IME), Rio de Janeiro 22290-270, Brazil; 3 Signal Processing Lab, COPPE/POLI, Technology Center, Federal University of Rio de Janeiro (UFRJ), Rio de Janeiro 21941-914, Brazil; seixas@lps.ufrj.br

**Keywords:** DoA estimation, time delay estimation, zero cyclic sum

## Abstract

Accurate direction of arrival (DoA) estimation is paramount in various fields, from surveillance and security to spatial audio processing. This work introduces an innovative approach that refines the DoA estimation process and demonstrates its applicability in diverse and critical domains. We propose a two-stage method that capitalizes on the often-overlooked secondary peaks of the cross-correlation function by introducing a reduced complexity DoA estimation method. In the first stage, a low complexity cost function based on the zero cyclic sum (ZCS) condition is used to allow for an exhaustive search of all combinations of time delays between pairs of microphones, including primary peak and secondary peaks of each cross-correlation. For the second stage, only a subset of the time delay combinations with the lowest ZCS cost function need to be tested using a least-squares (LS) solution, which requires more computational effort. To showcase the versatility and effectiveness of our method, we apply it to the challenging acoustic-based drone DoA estimation scenario using an array of four microphones. Through rigorous experimentation with simulated and actual data, our research underscores the potential of our proposed DoA estimation method as an alternative for handling complex acoustic scenarios. The ZCS method demonstrates an accuracy of 89.4%±2.7%, whereas the ZCS with the LS method exhibits a notably higher accuracy of 94.0%±3.1%, showcasing the superior performance of the latter.

## 1. Introduction

The precision and accuracy of an acoustic-based DoA estimation are essential across a spectrum of industries, spanning vital applications in both civilian and military sectors. Encompassing critical domains such as defense, law enforcement, security [1], and surveillance [2], reliable and precise DoA estimation ensures safety, strategic decision-making [3], and operational effectiveness. Applications such as gunshot DoA estimation [4], drone DoA estimation [5], and automotive angle estimation [6] require highly accurate estimates for optimal functionality.

The generalized cross-correlation algorithm (GCC) [7] is a relevant technique that estimates DoA; one of its most prominent advantages is the reduced computational effort enabled by the time delay estimation (TDE) [5] between pairs of sensors. Utilizing the cross-correlation function of the signals, the GCC algorithm facilitates an accurate determination of DoA, making it a highly reliable tool in spatial signal processing. Notably, its adaptability spans across various domains, including underwater acoustics [8], terrestrial acoustics [9], and radar [10] systems. The algorithm’s remarkable capability to handle diverse signal types and its resilience in noisy environments depend on the precision of the TDE process [5].

The TDE process, however, may be plagued by several factors that introduce errors, preventing an accurate DoA estimation [11]. A low signal-to-noise ratio (SNR) [12] constitutes one of the primary obstacles, as it weakens the discernibility of the signal of interest amidst background noise, leading to challenges in pinpointing the exact time delay of arrival. Furthermore, multipath propagation [13], a phenomenon where a signal arrives at the microphones through multiple paths, exacerbates the issue by causing time delay variations. This results in the reception of multiple, altered versions of the same signal, complicating the accurate identification of the original signal’s actual time of arrival. Additionally, errors in the measurement systems, including calibration inconsistencies (attitude and geometry of the array) [14] or hardware imperfections, further contribute to inaccuracies in time delay estimation, subsequently impacting the precision of DoA calculations. The cumulative impact of these factors on the acoustic signals leads to multiple peaks within the cross-correlation [5], resulting in misleading time delay estimations when considering only the peak of the cross-correlations with the highest amplitude (primary peak). Consequently, this multitude of peaks affects the accuracy of DoA estimation. Thus, addressing and improving this critical area in spatial signal processing is imperative for advancing the precision and reliability of DoA calculations.

The method proposed herein presents a framework that harnesses an underemphasized facet of the cross-correlation function: secondary peaks. By leveraging these secondary peaks, our approach contributes to the precision of DoA estimation, elevating it to new levels of accuracy. Our methodology circumvents the traditional constraints associated with time delay estimation by introducing a reduced complexity cost function. The first stage of this innovative two-stage method uses a low complexity cost function that integrates the ZCS condition, enabling its calculation for all delay combinations. The second stage comprises a more computationally complex least-squares cost function that demands more computational effort but with fewer delay combinations due to the results obtained with the first stage, i.e., a small set of delay vectors that minimize the ZCS cost function.

To illustrate the versatility of our approach, the method is applied to a challenging scenario involving the acoustic-based DoA estimation of drones, utilizing a four-microphone array setup. Our research underscores the potential of the proposed DoA estimation methodology through rigorous experimentation using both simulated and real-world data. This work propels the field of DoA estimation forward and has applications to near-real-time tasks.

Our contributions to the field of DoA estimation include the following: (1) recognizing the full potential of the ZCS cost function by computing all possible combinations of estimated delays; (2) mitigating computational complexity, allowing a thorough exploration of delay combinations to filter a concise set of candidate delay vectors; (3) identifying that the ZCS cost function may yield sub-optimal solutions to the DoA estimation problem; (4) acknowledging the potential for refinement of results by applying the LS solution; and (5) developing a DoA estimator system that requires minimal time and effort for users to set up and maintain, as it does not necessitate previous training.

This paper is organized as follows: Related work is presented in Section 2. Section 3 presents a brief overview of DoA estimation methods and the zero cyclic sum condition, followed by a discussion on the TDE problem. Section 4 explains the dataset and the preprocessing scheme. Section 5 details the proposed DoA estimation method. Section 6 presents the achieved results. Section 7 provides a discussion, while Section 8 concludes the paper.

## 2. Related Work

This section discusses works related to DoA estimation using different techniques, e.g., TDE-based techniques, beamforming, and AI-oriented DoA estimation methods.

### 2.1. Time-Delay Estimation Approaches

Reference [15] reviews the use of microphone arrays for sound sensing exploring the importance and limitations of ad-hoc microphones compared to other types. The contribution of this work is to establish a foundation for selecting the most suitable method for specific applications. A list of references on time delay estimation approaches can be found in [15].

Reference [11] focuses on gunshot DoA estimation when the SNR is low. It combines GCC-PHAT, exhaustive search, and the search for a fundamental loop. The method searches for the best set of microphone pairs. It makes a partial scan across the primary and secondary peaks of the cross-correlations (due to the computational efforts and number of microphones used).

The work highlighted in [16] explores simultaneous sound source localization (SSL), a critical study area. It navigates the balance between low computational complexity and high accuracy in SSL algorithms by combining a one-step-based method using generalized eigenvalue decomposition and a two-step method employing adaptive generalized cross-correlation with phase transform/maximum likelihood filters. This technique, complemented by a unique T-shaped circular distributed microphone array, aims to enhance 3D multiple simultaneous SSL. The 3D location of each acoustic source is estimated by finding the closest point to all estimated DOAs.

The work outlined in [17] addresses the challenge of noise and reverberation in time difference of arrival (TDOA) estimation. This research introduces two methods to estimate TDOA in environments affected by noise and reverberation effectively. The proposed methods leverage the linear phase structure observed across frequencies in a steering vector and capitalize on its absolute phases to mitigate potential noise and mathematical complications. By transforming the TDOA estimation into an optimization problem solvable via Newton’s method, the study presents experimental evaluations in simulated acoustic settings. Their fast-search method demonstrates superior TDOA accuracy and computational efficiency in environments with moderate-to-high input SNR and low reverberation.

References [18,19] focus on sniper detection, utilizing audio signals from gunshot recordings via a microphone array. Reference [18] employs the GCC-PHAT algorithm for DoA estimation, revealing that time lags between the two largest peaks in the correlation functions align with the muzzle blast and shockwave components. While the phase transform method excels in peak separation, the study concludes that muzzle blast DoA estimation based on the maximum correlation peak obtained by other GCC techniques is generally more accurate.

Reference [20] addresses the task of gunshot DOA estimation, crucial for enhancing public and troop safety. The proposed algorithm is designed for scenarios with highly noisy signals, which commonly occur in sniper situations where the firing position is distant from the sensor array. In such scenarios, signal-to-noise ratio reduction poses a challenge to accurate DOA estimation. The paper introduces an innovative approach that combines an exhaustive search for optimal microphone pairs in the array, aiming for superior DOA estimation results and rapid response times across various shooting scenarios. The focus is particularly on highly corrupted signals where existing algorithms may fail. The proposed scheme is evaluated using experimental data from both simulated and recorded gunshot signals. Another application used to estimate the DoA of gunshots—using the same algorithms but with the microphone array embedded in a drone—can be seen in [4,9,21,22].

The research on acoustic-based drone DoA estimation carried out in [5] addresses the challenge of accurately estimating the DoA of a drone in acoustically complex environments using a seven-microphone array. The focus is on improving TDE from a set of time delay candidates, particularly when dealing with strongly corrupted audio signals affected by noise and multipath. The traditional approach faces difficulties in accurately estimating TDE without relying on a line-of-sight assumption. The proposed solution utilizes genetic algorithms to perform a heuristic search for correct delays among possible pairs of microphones. A fitness function based on the concept of ZCS of closed loops is introduced, ensuring that the sum of theoretical delays in a closed loop equals zero. Experimental results, both in simulations and real-world trials, demonstrate the method’s effectiveness in identifying correct delays, demonstrating its potential for practical drone DoA estimation in challenging acoustic environments.

Reference [23] implements a detection fusion algorithm and a TDOA estimation algorithm grounded in Bayesian filtering principles. This study employs two acoustic arrays, each comprising four microphones with tetrahedron shapes. Reference [24] proposes innovative methods to mitigate the detrimental effects of reverberation on audio source localization. By incorporating models for both early reflections and the audio source itself, the authors introduce two iterative approaches for estimating the DOA of both the direct path and early reflections. The early reflections are effectively subtracted from the signal observations before localizing the direct path component, which reduces bias. Simulation results demonstrate the efficacy of these techniques, showcasing more accurate DOA estimation compared to state-of-the-art methods in both synthetic and real-world scenarios with reverberation.

Reference [25] addresses the challenge of estimating the DOA of incident plane waves in scenarios where phase noise corrupts the received data (besides other additive noise). The proposed methodology adopts a Bayesian framework and employs a variational mean-field approximation to account for phase noise. By integrating sparse-enforcing distribution priors on DOA and Markov model priors on phase noise, the novel algorithm demonstrates superior performance compared to conventional beamforming and similar variational approaches with non-informative priors. Simulation results underscore the efficacy of the proposed approach in accurately estimating DOA amidst phase noise corruption. Future research directions will extend this investigation to underwater acoustics, further elucidating the algorithm’s applicability in real-world environments.

In [26], a constrained least squares estimator is developed for estimating the azimuth and elevation of a sound emitter in three-dimensional space using TDOA measurements obtained from an array of acoustic sensors. Addressing scenarios where the source emits transient signals, necessitating reliance solely on TDOA measurements for direction finding, the study highlights limitations of conventional linear least squares estimators due to inherent information loss during the linearization of nonlinear observation equations. To mitigate this issue, the paper proposes a constrained least squares estimator that leverages both Lagrange multiplier and quadratic constraints to formulate the cost function. The resulting estimator offers an approximate closed-form solution, significantly reducing computational complexity while maintaining high accuracy. Through simulation and field experimental validation, the proposed method demonstrates superiority over traditional linear and nonlinear estimators, demonstrating its potential for robust and efficient direction finding in practical applications.

Reference [27] introduces a novel approach for distributed acoustic tracking by incorporating the coherent-to-diffuse ratio (CDR) as a measure of DoA reliability. Utilizing the CDR as the concentration parameter in the DoA-likelihood function—modeled by a von Mises distribution—enables the tracking of source positions over time at individual nodes using a von Mises filter. By evaluating the von Mises filter for a range of uninformative range hypotheses, the method leverages network fusion to exploit spatial diversity among nodes, probabilistically triangulating the relevant source positions and range hypotheses. Realistic simulation results demonstrate significant improvements over classical approaches, enhancing accuracy by up to 39% compared to constant concentration parameter methods and up to 74% compared to least-squares source triangulation techniques [21].

### 2.2. Beamforming Techniques

Reference [28] provides a focused analysis of common beamforming algorithms, presenting both theoretical insights and recent applications in real cases. Rather than a broad exploration, the emphasis is on harmonizing the sector through a combined approach. The goal is to offer a resource for academics seeking theoretical understanding and technicians selecting algorithms for varied measurement conditions. With a lack of comparative studies in the literature, the authors address this gap, advocating for research in algorithm performance in similar scenarios. While acknowledging the limitations of certain algorithms, the work generally recommends deconvolution algorithms (CLEAN-SC, DAMAS) or MUSIC for acoustic camera users due to their accuracy, even though they are slower and more complex. The authors propose a combination of algorithms for research purposes, anticipating future implementations in commercial acoustic camera software.

Reference [29] introduces the use of delay-and-sum to enhance sniper positioning estimates. The delay-and-sum beamforming is used for improved detection of shockwave and muzzle blast acoustic signatures. The approach not only enhances the signal-to-noise ratio—doubling the detection range for a four-microphone array—but also demonstrates robustness in handling single- and multi-shot events and reflections, contributing to more reliable sniper location estimation. Other contributions to DoA estimation using delay-and-sum techniques can be found in [30,31].

Reference [32] investigates DoA for multiple acoustic sources using the approximate maximum likelihood (AML) algorithm. This algorithm facilitates the estimation of DoAs through an iterative search process, demonstrating versatility in both 2D and 3D scenarios. By employing blind beamforming techniques, the study showcases the capability of the AML algorithm to estimate azimuth angles for sources in the far field of the array, as well as azimuth and elevation angles. The authors provide comprehensive analyses, including the calculation of Cramér–Rao bound (CRB) on DoA estimation, and introduce the concept of an isotropic array to enhance accuracy across the spatial domain. Simulation and experimental results validate the performance of the 3D AML algorithm in scenarios involving multiple sources at varying azimuth and elevation angles.

Reference [33] proposes an innovative approach to address the DOA estimation challenge within acoustic environments utilizing microphone arrays. The method initially transforms the received noisy speech signals into the STFT domain. Subsequently, a Householder transformation is constructed and applied to the multichannel STFT coefficients, segregating them into components dominated by the signal of interest and noise. By forming a cost function from the transformed coefficients, the method facilitates the extraction of DOA information by searching for extremum values within the angle range between 0 and 180 degrees. Simulation results presented in the paper demonstrate the effectiveness of this approach in achieving accurate DOA estimation.

In [34], the authors advocate an in-depth exploration of various algorithms, such as Bartlett beamforming, Capon beamforming, eigenvector, and the acoustic intensity vector for the DoA estimation of both single and multiple sources employing an L-shaped acoustic vector sensor (AVS). This specialized AVS configuration integrates three homogenous sensors, each comprising omnidirectional microphones with a 14.14 mm aperture. To facilitate experimental signal recording within the L-shaped AVS environment, the authors employ COMSOL Multiphysics, leveraging its finite element method capabilities. Through systematic investigation and comparative analysis, the study offers valuable insights into the efficacy and performance nuances of different DoA estimation algorithms within the context of the L-shaped AVS configuration.

Reference [35] proposes a calibration procedure for custom 3D AVS tailored for accurate DoA estimation. This calibration method addresses amplitude and phase differences among sensor components, crucial for precise DoA computation. Through experimental validation using low-cost MEMS microphones and DSP boards, the proposed procedure matches the accuracy of high-cost, factory-calibrated sensors. The study underscores the applicability of the calibration algorithm in practical scenarios such as environmental and traffic monitoring, offering a cost-effective solution for reliable sound source localization. Further research is suggested to expand the evaluation scope and refine the calibration approach for broader deployment.

Reference [36] introduces a novel approach for multiple source DOA estimation using the maximum likelihood method in the spherical harmonic domain. By employing an efficient sequential iterative search of maxima on the cost function, the proposed method achieves superior performance compared to traditional beamformer-based and subspace-based methods. Notably, the method avoids the computational burden associated with high-dimensional grid search, making it suitable for both rigid-sphere and open-sphere configurations. Simulation and experimental validations conducted in various acoustic environments demonstrate the effectiveness and stability of the proposed method, highlighting its potential for practical applications in room geometry inference, source separation, and speech enhancement.

### 2.3. AI-Oriented DoA Estimation

Reference [37] reviews the cutting-edge applications of artificial intelligence (AI) in the domain of beamforming. Through a comprehensive exploration of AI-centric beamforming studies, the work aims to elucidate and extract meaningful insights into the role of AI in enhancing beamforming performance. Beginning with an overview of beamforming and its adaptive algorithms, as well as DOA estimation methods, the analysis explores machine learning classes, basic neural network topologies, and efficient deep learning schemes. The paper further explores the optimal utilization of ML and NNs, both independently and in conjunction with other applications such as ultrasound imaging, massive multiple-input multiple-output structures, and intelligent reflecting surfaces. Special emphasis is placed on the realization of beamforming or DOA estimation setups through deep learning topologies. Concluding with significant insights and a discussion on prospects and research challenges, the survey provides a comprehensive overview of the evolving landscape of AI in beamforming.

Reference [38] explores deep learning-based time-frequency masking to enhance TDOA estimation in challenging noisy and reverberant environments. Three algorithms are introduced to fortify conventional methods used for speaker localization, utilizing deep neural networks to identify cleaner time-frequency units for more accurate TDOA estimation. These algorithms exhibit robustness in scenarios with low SNR, high reverberation, and a low direction-to-reverberant energy ratio.

Reference [39] introduces a novel high-resolution beamforming method employing genetic algorithms. By leveraging the sparsity of acoustic sources, the approach reconstructs the source vector through optimization within a sound propagation model. To enhance efficiency, the algorithm narrows down the search domain through prior correlation analysis. Numerical and experimental comparisons with conventional beamforming methods demonstrate the superior accuracy and robustness of the proposed genetic algorithm beamforming. Breaking through resolution limits, it accurately recovers the distribution of acoustic sources in two- and three-dimensional spaces.

Reference [40] presents a cost-effective small UAS acoustic detection system utilizing a four-microphone array that estimates DOA and UAS identification via machine learning techniques. Extensive outdoor experiments validate its efficacy in reliably detecting UAS at distances exceeding 70 m, offering enhanced situational awareness of surrounding airspace. Future research aims to address DOA angle fluctuations in real-time experiments and explore enhancements for UAS identification, including diverse sound sample collection and neural network architecture exploration.

In [41], the authors advocate a learning-based approach for DOA from microphone array input, addressing limitations inherent in traditional signal processing methods like the classic LS method. These conventional techniques are constrained by stringent assumptions on signal models and require precise estimations of TDOA, making them susceptible to noise and reverberation distortions. By contrast, the proposed learning-based approach leverages a multilayer perceptron neural network to learn from extensive simulated noisy and reverberant microphone array inputs, enabling robust DOA estimation. Extracting features from GCC vectors, the model effectively captures the nonlinear mapping to the DOA. Notably, the method’s accuracy improves with the availability of more training data. Experimental evaluations on both simulated and real data demonstrate significant performance gains over the state-of-the-art LS method, with reduced root-mean-square error (RMSE) particularly evident in real-world scenarios such as meeting rooms.

In [42], the authors advocate a novel CNN approach for broadband DOA estimation, wherein the phase component of short-time Fourier transform coefficients from microphone signals serves as direct input to the CNN. During training, the network autonomously learns the requisite features for accurate DOA estimation. Leveraging only the phase component of input facilitates training with synthesized noise signals, simplifying the dataset preparation compared to utilizing speech signals. Experimental assessments validate the framework’s capability to generalize to speech sources and its robustness to noise, minor microphone position perturbations, and diverse acoustic conditions. Through both simulated and real data experiments, the study underscores the CNN’s adaptability and resilience, signaling the promising potential for practical DOA estimation applications.

### 2.4. Partial Conclusions

According to the literature review, GCC-PHAT performs well at localizing acoustic sources even in reverberant and noisy environments, making it well-suited for the often complex acoustic conditions associated with outdoor drone detection scenarios. Beamforming algorithms involve complex mathematical operations. In scenarios with real-time processing requirements, the computational load can become prohibitively high, leading to delayed DoA estimations and resource-intensive operations.

Based on the characteristics of the methods investigated herein, the GCC-PHAT method emerges as the technique to explore for estimating drone DoA using acoustics, owing to its potential for use in adverse environments and its lower computational cost. In addition, there is a gap in the literature that can unleash the potential of GCC-PHAT in highly noisy environments: the utilization of secondary peaks in the cross-correlations.

## 3. DoA Estimation Techniques

This section details the DoA estimation techniques employed in this work.

### 3.1. GCC-PHAT (Classic DOA Estimation)

The foundation of the GCC method lies in the utilization of the cross-correlation function, which gauges the resemblance between two signals concerning their time delay [7]. The GCC method computes the cross-correlation function between pairs of microphone signals in order to derive the TDE between these signals. The basic principle is to find the time delay that maximizes the cross-correlation function, $r_{x_{i}x_{j}}\left(\tau \right)$, defined as follows [7]:

(1) $$r_{x_{i}x_{j}}\left(\tau \right)=E\left[x_{i}\left(k\right)x_{j}\left(k-\tau \right)\right],$$

where E[·] is the expectation operator and τ is the delay (in number of samples) between the signals of two given sensors, $x_{i}\left(k\right)$ and $x_{j}\left(k\right)$. By identifying peaks within the cross-correlation function, potential time delay values between the signals are discerned, offering a basis for determining the DoA of a given sound source.

The GCC is usually obtained as follows:

(2) $${\hat{r}}_{x_{i}x_{j}}^{\mathrm{GCC}}\left(\tau \right)=\frac{1}{2\pi }\int_{-\pi }^{\pi }\psi \left(\omega \right)X_{i}\left(e^{j\omega }\right)X_{j}(e^{j\omega )}\;d\omega \;,$$

where ψ(ω) is the GCC-PHAT method that normalizes the magnitude spectrum of the cross-correlation function through the following process:

(3) $$\psi^{\mathrm{PHAT}}\left(\omega \right)=\frac{1}{|X_{i}\left(e^{j\omega }\right)X_{j}\left(e^{-j\omega }\right)|},$$

such that [7]

(4) $${\hat{r}}_{x_{i}x_{j}}^{\mathrm{PHAT}}\left(\tau \right)=\frac{1}{2\pi }\int_{-\pi }^{\pi }\frac{X_{i}\left(e^{j\omega }\right)X_{j}(e^{j\omega )}}{|X_{i}\left(e^{j\omega }\right)X_{j}\left(e^{j\omega )}\right|}\;d\omega \;.$$

Finally, the TDE is obtained as follows:

(5) $${\hat{\tau }}_{ij}=\underset{\left|\tau \right|\leq \tau_{\max}}{arg max}\left|{\hat{r}}_{x_{i}x_{j}}^{\mathrm{PHAT}}\left(\tau \right)\right|,$$

where $\tau_{\max}$ represents the highest achievable delay, determined by the spatial separation between microphones *i* and *j*. Additionally, a regularization term in the denominator of $\psi^{\mathrm{PHAT}}\left(\omega \right)$ can be used to avoid division by zero.

This normalization procedure efficiently improves the phase information while reducing the disparities in signal amplitudes, leading to enhanced accuracy in the estimation of a time delay. By incorporating phase information, the GCC-PHAT method attains increased resilience against reverberation and noise in contrast to the original GCC method [7].

Upon deriving the delay estimations, it is possible to estimate the unit norm vector $a_{\mathrm{DoA}}$ (pointing toward the DoA) by minimizing the LS cost function, i.e.,

(6) $$\xi =\sum_{i=0}^{k}{\left(\hat{\tau }-\Delta p_{ij}^{T}a_{\mathrm{DoA}}\right)}^{2},$$

with respect to the closed-form LS solution, as follows:

(7) $$a_{\mathrm{DoA}}=R^{-1}p,$$

where the 3×3 matrix **R** is given by the following:

(8) $$R=\Delta p_{12}\Delta p_{12}^{T}+\Delta p_{13}\Delta p_{13}^{T}+\cdots +\Delta p_{(N-1)N}\Delta p_{(N-1)N}^{T},$$

$\Delta p_{ij}$ with $p_{i}-p_{j}$, $p_{i}$, and $p_{j}$ corresponding to the positions of sensors *i* and *j*, and the 3×1 vector **p** is given by the following:

(9) $$p={\hat{\tau }}_{12}\Delta p_{12}+{\hat{\tau }}_{13}\Delta p_{13}+\cdots +{\hat{\tau }}_{(N-1)N}\Delta p_{(N-1)N}.$$

Vector $a_{\mathrm{DoA}}=R^{-1}p$ readily provides the direction of arrival, encompassing both azimuth (the horizontal angle ϕ) and zenith (the vertical angle θ, complement of the elevation) [11].

Another critical function in the GCC-PHAT method involves interpolation, which serves to improve the accuracy and precision of time delay estimation. Through the application of interpolation methods, the GCC-PHAT method can achieve more precise time delay estimations and effectively manage sub-sample time delay resolutions [4]. This technique is only effective in refining the precision of a DoA estimation from the correct peaks.

### 3.2. TDE Problem

A frequent error encountered in time delay estimation based on cross-correlation arises from the existence of noise. When noise affects the correlated signals, it has the potential to introduce false correlations, causing inaccurate time delay estimations. This noise can distort the cross-correlation function, thereby causing misleading peak positions or the appearance of false peaks with high amplitudes that do not align with the actual time delay of the signal of interest.

Inaccurate time delay estimation can also stem from strong reverberation or multipath effects within the received signals. The presence of reverberation markedly impacts the form and strength of the cross-correlation function, complicating the precise identification of the genuine peak denoting the direct path time delay. Sound’s reflections and diverse pathways may generate extra peaks or alter the primary peak, resulting in erroneous estimations.

**Figure 1:**
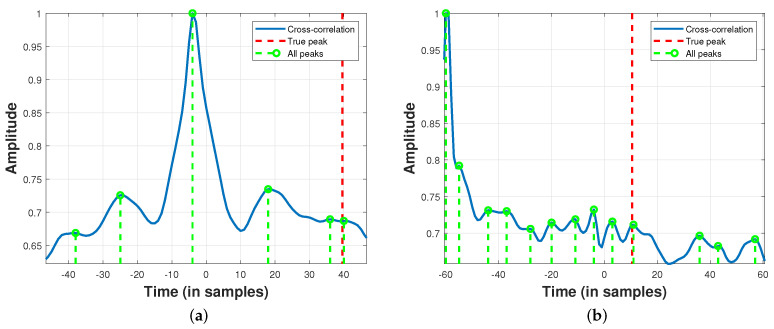
Cross-correlations of acoustic signals collected from a Phantom 4 drone hovering in an outdoor environment: (**a**) 5th peak corresponds to the correct time delay; (**b**) 9th peak corresponds to the correct time delay.

Figure 1 illustrates pertinent issues associated with TDE when the SNR is low. In Figure 1a, the accurate TDE in the fifth peak, sorted by descending amplitude order, should be noted. Figure 1b showcases a worse case in which only the ninth peak corresponds to the correct time delay. In conclusion, the cross-correlations encapsulate the requisite information for accurate DoA estimation. However, the efficacy of these estimations is compromised by the low SNR of the target signal.

The multitude of distinct peaks in the cross-correlations related to drone noise presents intriguing opportunities for simulations and experimental investigations. These opportunities involve exploring the secondary peaks, leveraging interpolated peaks to enhance estimation precision, and comparing them with classical DoA estimation methods that solely consider the central peak of the cross-correlations. A comprehensive exploration of these aspects can contribute to an improved understanding and the refinement of TDE methodologies in real-world scenarios.

### 3.3. Impact of TDE Error in DoA Estimation

**Figure 2:**
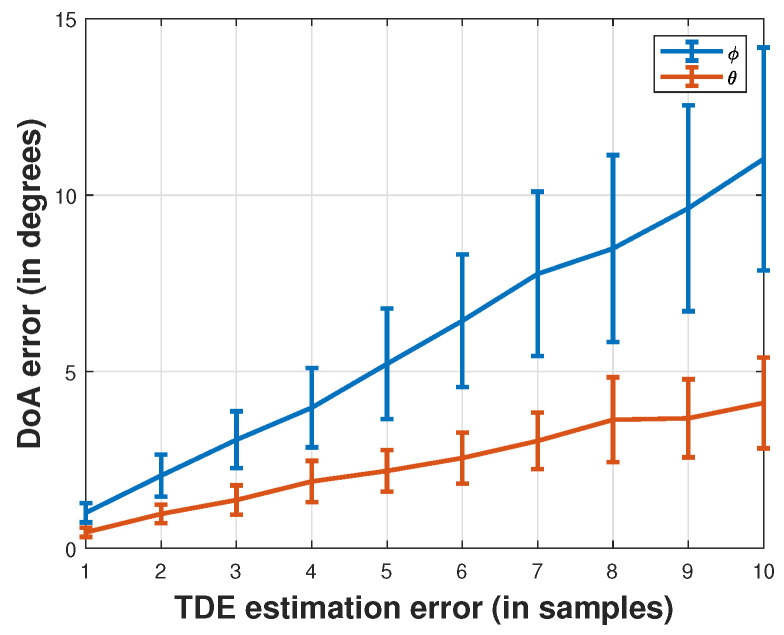
Error of DoA according to the TDE additive noise.

Figure 2 depicts the relationship between the DoA error and TDE error, providing valuable insights into the accuracy of the localization process. The graph illustrates that as the TDE estimation error increases, there is a corresponding rise in the DoA error, indicating a direct correlation between the two parameters. The analysis reveals that TDE errors within a maximum range of three samples remain acceptable, as they correspond to DoA errors of less than 5 degrees for zenith and azimuth angle estimations. This observation underscores the robustness of the localization system, suggesting that minor deviations in TDE estimation do not significantly compromise the accuracy of DoA predictions within a reasonable margin.

### 3.4. ZCS Condition

The ZCS condition is obtained with a set of microphones that forms a closed loop. The theoretical time delays obtained by the spatial distance between each pair of microphones that forms a closed loop is an abelian group, i.e., a commutative set of delays. The sum of all elements of an abelian group is known to be 0, regardless of the order in which they are added [43]. This criterion finds practical application in digital signal processing within an array of sensors [5].

## 4. Dataset and Preprocessing

This research focuses on using drone noise as the signal of interest for experimental tests aimed at estimating the DoA. The choice of drone noise as the experimental input evaluates the method under conditions that are of great interest to defense forces, law enforcement, and surveillance agencies.

### 4.1. Data Acquisition

**Figure 3:**
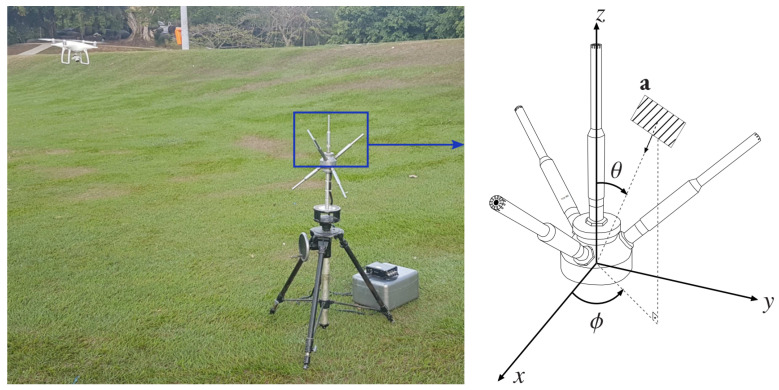
Drone data acquisition setup featuring a close-up view of the four upward-pointing microphones employed for signal collection. Note the unit vector in the direction of the wave’s front: $a=-{\left[\sin\theta \cos\phi \;\;\sin\theta \sin\phi \;\;\cos\theta \right]}^{T}$, where ϕ corresponds to the azimuth and θ to the zenith.

Figure 3 illustrates the Phantom 4 drone (DJI, Shenzhen, China), an array of Behringer ECM8000 [44,45] microphones (Willich, Germany), and the Zoom F8 recorder (Hauppauge, NY, USA), the latter serving to convert analog signals into digital format. The data acquisition process involves capturing acoustic signals emitted by the drone and background noise. Following this signal acquisition, the dataset undergoes comprehensive offline analysis to extract valuable insights and make well-founded assessments.

### 4.2. Acoustic Drone Noise Analysis

**Figure 4:**
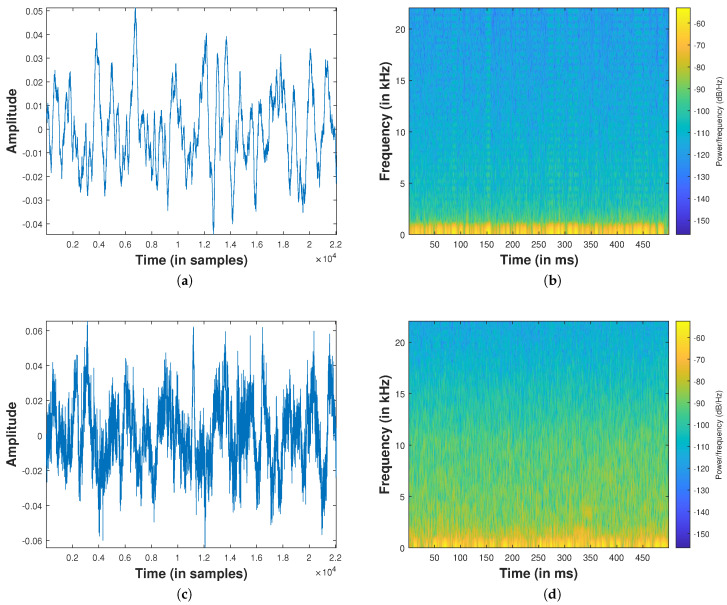
Background noise and drone noise 10 m away from the microphone: (**a**) Background noise time domain, (**b**) spectrogram background noise; (**c**) drone noise time domain, (**d**) drone noise spectrogram.

Figure 4 depicts both the background noise and the signals emitted by the hovering Phantom 4 drone, illustrating a duration of 500 ms. The figure also includes a spectrogram computed with a sample rate of 48 kHz. Notably, in optimal conditions and when the drone is near the microphone array, it becomes feasible to capture drone noise in higher frequency ranges, extending up to 13.5 kHz.

For a more comprehensive exploration of the acoustic characteristics of drone noise, interested readers are encouraged to refer to the extensive study presented in [46,47,48]. These references analyze drone noise and provide valuable insights into the subject matter.

## 5. Proposed Method

In the initial stage of the ZCS-LS method, an exhaustive search is performed, calculating the ZCS cost function of all possible combinations of TDE. This computation identifies a small set of candidate time delay vectors characterized by the lowest ZCS cost function values. Transitioning to the second stage, one vector from this set is selected. This selection process is guided by the LS cost function, ensuring that the chosen candidate time delay vector possesses one of the lowest ZCS cost functions among all possible time delay vectors and aligns optimally with the LS solution. By systematically navigating through these stages, the ZCS method reduces the complexity of obtaining an accurate DoA estimate that considers the cross-correlations’ primary and secondary peaks. At the same time, the use of the LS cost function enhances the DoA estimation performance.

### 5.1. Exhaustive Search with ZCS

For an exhaustive search, careful consideration is given to the number of microphones in the array. The selection of a four-microphone array over a larger array, such as a seven-microphone configuration, is grounded in computational feasibility and efficiency. The restriction to four microphones allows a manageable number of microphone pairs, specifically N=M2. For each pair of microphones, we window the signal and apply the cross-correlation function to estimate the time delay. In this method, primary and secondary peaks of the cross-correlations are taken into account, achieving (*C*) candidate time delays for each cross-correlation.

The method explores the entire solution space, denoted as *S*, which is the set of all possible time delay combinations $S=C^{N}$ within a four-microphone array (M=4), considering (*C*) time delay candidates for each of the six cross-correlations of N=42=6. This choice ensures a systematic evaluation of feasible delay combinations. Ten is considered the maximum delay to encompass the widest range of the rightmost peak in the signal.

In contrast, the computational complexity grows exponentially with an increase in the number of microphones. For instance, in a seven-microphone array, denoted by M=7, the solution space, denoted as *S* with *C*, representing the set of possible choices of delays estimated using the cross-correlation function, is determined by $S=C^{N}$, where N=21 for M=7 microphones. If C=2, the solution space is $C^{2}$ = 2,097,152. As *C* increases, the solution space expands rapidly. When C=3, the solution space grows to a massive 10,460,353,203 potential solutions, and for C=4, it reaches 4,398,046,511,104 possible solutions, representing trillions of unique combinations. This exponential growth underscores the practical advantages of utilizing a four-microphone array, ensuring computational efficiency in exploring the solution space for optimal DoA estimation.

The enormous solution space for a four-microphone array constrains the cost function used in the exhaustive search process. This is where the reduced complexity cost functions using ZCS excel. By applying a low complexity cost function, the exhaustive search can efficiently explore these expansive solution spaces and navigate toward the global optimal solution.

Each time delay combination is evaluated based on the ZCS cost function that quantifies their proximity to a zero-sum. The closer to a zero-sum, the more coherent the time delays for a given DoA. The ZCS method can efficiently identify the correct delays from a multitude of incorrect delays, particularly in situations with low SNR. The (*C*) candidate delays for each cross-correlation function, $r_{x_{i}x_{j}}$, are the elements of each row of the data matrix, V. For M=4, which results in N=6, the N×C matrix V with all candidate delays is defined as follows:

(10) $$V=\left[\begin{array}{ccccc} {\hat{\tau }}_{12,1} & {\hat{\tau }}_{12,2} & {\hat{\tau }}_{12,3} & \cdots & {\hat{\tau }}_{12,C}\\ {\hat{\tau }}_{13,1} & {\hat{\tau }}_{13,2} & {\hat{\tau }}_{13,3} & \cdots & {\hat{\tau }}_{13,C}\\ {\hat{\tau }}_{14,1} & {\hat{\tau }}_{14,2} & {\hat{\tau }}_{14,3} & \cdots & {\hat{\tau }}_{14,C}\\ \vdots & \vdots & \vdots & \vdots & \vdots \\ {\hat{\tau }}_{34,1} & {\hat{\tau }}_{34,2} & {\hat{\tau }}_{34,3} & \cdots & {\hat{\tau }}_{34,C} \end{array}\right],$$

such that each column corresponds to each possible combination of delays, denoted as the N×1 vector $v_{c},1\leq c\leq C$.

The ZCS cost function [5] plays a pivotal role in this method, assessing the sum of delays in defined subsets that form closed loops to minimize instances of inaccurate zero-sum outcomes. By comprehensively examining all potential subsets that form closed loops, and summing their results, the method effectively diminishes the probability of encountering a zero-sum outcome that lacks the correct delays.

To facilitate the computational calculation of the ZCS cost function, a method was devised to identify and enumerate closed loops based on the number of delays. More specifically, when employing an M=4 microphone array, we find that, with three delays, there are four closed loops, and with four delays, one closed loop. In this context, the total number of closed loops, denoted as *L*, corresponds to L=4+1=5. It is important to note that delay, $\tau_{31}$, which closes the loop, can be determined by taking the negative value of $\tau_{13}$. Similarly, $\tau_{43}$ can be expressed as $-\tau_{34}$, and in general, any delay, $\tau_{ji}$, that closes the loop can be written as $\tau_{ji}=-\tau_{ij}$. By utilizing this property, it is possible to compute all possible delays and then manipulate them to identify the correct value of τ, which closes the loop. This approach saves computational resources by avoiding redundant calculations and facilitates the determination of the correct delay for loop closure. The complete listing of all possible closed loops for three and four delays can be found in Table 1.

Based on Table 1, we create an L×N matrix D, such as for M=4:

(11) $$D=\left[\begin{array}{cccccc} 1 & -1 & 0 & 1 & 0 & 0\\ 1 & 0 & -1 & 0 & 1 & 0\\ 0 & 1 & -1 & 0 & 0 & 1\\ 0 & 0 & 0 & 1 & -1 & 1\\ 1 & 0 & -1 & 1 & 0 & 1 \end{array}\right];$$

such that, for a given choice of delays in v, each element of vector f=Dv corresponds to the sum of all subsets (closed loops). The ZCS cost function is then calculated as follows:

(12) $$f=f^{T}f={∥f∥}^{2}.$$

This ZCS cost function captures the squared norm of the resulting vector f, encompassing the contributions from all subsets and providing a measure of the coherence among the time delays concerning an arbitrary DoA.

The best *Z* vectors, determined by the ascending order of ZCS scores, are stored and form a collection of potential solutions to the problem achieved with low computational effort. Each combination of time delays serves as a plausible solution to the DoA estimation problem represented as a column within matrix $P_{N\times Z}$:

(13) $$P=\left[\begin{array}{ccccc} {\hat{\tau }}_{11} & {\hat{\tau }}_{12} & {\hat{\tau }}_{13} & \cdots & {\hat{\tau }}_{1Z}\\ {\hat{\tau }}_{21} & {\hat{\tau }}_{22} & {\hat{\tau }}_{23} & \cdots & {\hat{\tau }}_{2Z}\\ {\hat{\tau }}_{31} & {\hat{\tau }}_{32} & {\hat{\tau }}_{33} & \cdots & {\hat{\tau }}_{3Z}\\ \vdots & \vdots & \vdots & \vdots & \vdots \\ {\hat{\tau }}_{N1} & {\hat{\tau }}_{N2} & {\hat{\tau }}_{N3} & \cdots & {\hat{\tau }}_{NZ} \end{array}\right],$$

while matrix $P_{N\times Z}$ presents a range of potential solutions, it is essential to note that the time delay vector with the lowest ZCS score may not always constitute the optimal combination for accurate DoA estimation. Consequently, a second phase is introduced to refine the ultimate selection from the pool of *Z* candidate vectors further. This additional step aims to enhance the precision and reliability of the chosen solution, ensuring that the DoA estimation is not solely dependent on the ZCS score but also considers an additional LS cost function for a more precise outcome.

### 5.2. Least-Squares Cost Function

This extra method aims to enhance accuracy and efficiency in DoA estimation, ensuring that the selected delays contribute significantly to the DoA estimated. It consists of estimating the DoA with a given time delay vector and analyzing the sum of the squared error of the time delay calculated according to the DoA and each original time delay.

### 5.3. Summary

The ZCS-LS method is detailed in Algorithm 1.
**Algorithm 1** Exhaustive search using ZCS and LS.// Compute all (*C*) candidate delays for every $r_{x_{i}x_{j}}$:**for **i=1:N **do**   Compute ${\hat{r}}_{x_{i}x_{j}},ij=12\;\;\mathrm{to}\;\;34$   Obtain (*C*) candidate delays (larger peaks of ${\hat{r}}_{x_{i}x_{j}}$)   $V_{i,:}⇐\left[{\hat{\tau }}_{ij,1}\;\;{\hat{\tau }}_{ij,2}\;\;\cdots \;\;{\hat{\tau }}_{ij,C}\right]$**end for**// Create a combination of time delays and compute ZCS:**for** i=1:S **do**   $P_{:,i}⇐$ map ${\hat{\tau }}_{ij,i}$ in $V_{i,:}$   $f=f^{T}f={∥f∥}^{2}$   $P_{N+1,i}=f$**end for**// Compute LS cost function of the *Z* time delay vectors with the lowest ZCS:**for** i=1:Z **do**   $\xi =\sum_{i=0}^{k}{\left(\hat{\tau }-\Delta p_{ij}^{T}a_{\mathrm{DoA}}\right)}^{2}$   $P_{N+2,i}$ = ξ**end for**// Choose the time delay vector with the lowest ξ (LS cost function):$v=P_{1:N,i}=arg min\xi$

## 6. Results

### 6.1. Effects of Signal Window Length

**Figure 5:**
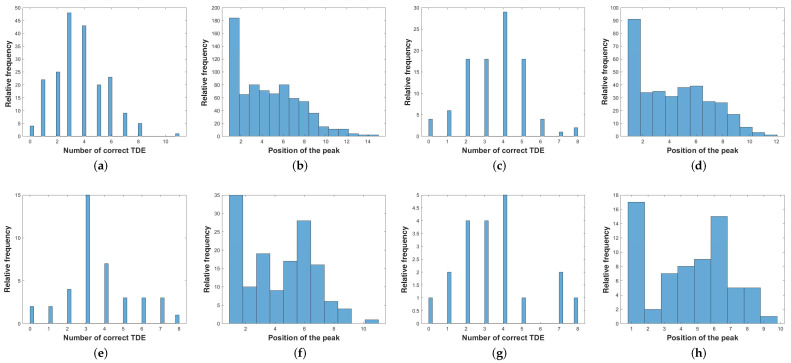
The statistics of the cross-correlations $r_{x_{i}x_{j}}$. They reveal the number of accurately estimated delays (comprising all peaks from $r_{x_{i}x_{j}}$ and considering ±3 samples error) within distinct time windows of 100 ms, 200 ms, 500 ms, and 1000 ms for cases (**a**,**c**,**e**,**g**), respectively. In addition to the number of accurately estimated delays, the statistics of the cross-correlations $r_{x_{i}x_{j}}$ provide insights into the peak position based on the descent amplitude criterion within specific time windows of 100 ms, 200 ms, 500 ms, and 1000 ms for cases (**b**,**d**,**f**,**h**), respectively.

Before conducting simulations, a comprehensive evaluation of the actual signals was undertaken to achieve a more faithful emulation of real-world conditions. This experimental assessment is imperative to understand the frequency with which primary and secondary peaks accurately indicate the actual time delay. Figure 5 illustrates the histogram detailing the position of the actual time delay. Specifically, it showcases the number of peaks sorted in amplitude descending order, highlighting instances where the correct time delay is successfully retrieved (with a permissible error of ±3 samples). This scrutiny serves as a foundational step in ensuring the potential to explore the secondary peaks to estimate the DoA.

### 6.2. DoA Estimation with Simulated Data

**Figure 6:**
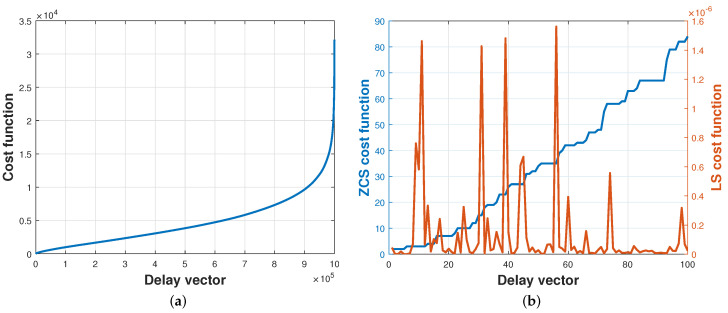
Evolution ZCS and LS cost functions. (**a**) ZCS cost function computed for all possible combinations of delays and (**b**) ZCS and LS cost functions computed for the first 100 combinations that minimize ZCS. The delay vector that minimizes the ZCS cost function is not the one that minimizes the LS cost function.

To run simulations, we created simulated V matrices according to $\tau_{max}$ between each pair of microphones. Figure 6 describes the cost function computed with simulated data. In Figure 6a, all delay combinations are calculated using ZCS and sorted in ascending order according to the ZCS cost function. Figure 6b depicts the first Z=100 ZCS and LS cost function using actual data.

**Figure 7:**
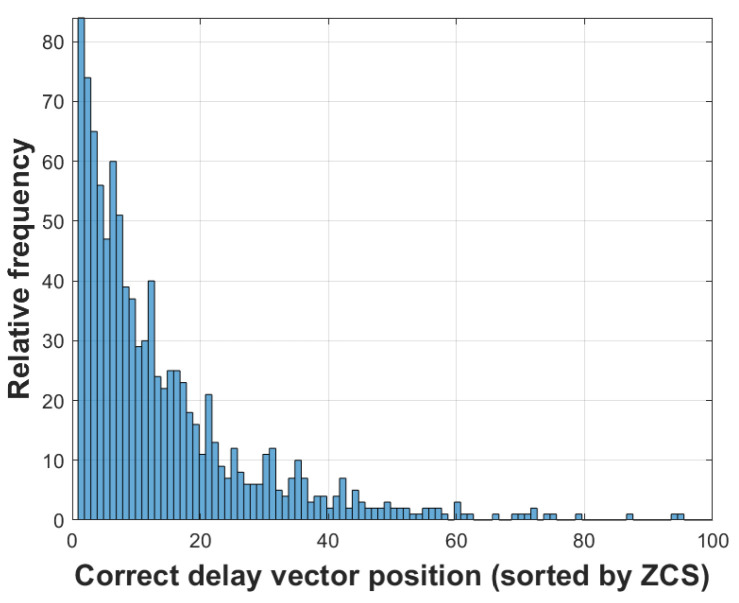
Histogram of the position of the correct set of delays (1000 independent trials).

The ZCS cost function facilitates the computation of all *S* potential combinations of time delays presented in matrix V. Figure 7 illustrates 1000 runs with simulated data, displaying the position at which the correct time delay vector is situated according to the ZCS. Although the ZCS itself does not determine the optimal combination of time delays, Figure 7 illustrates that this cost function effectively places the correct time delay vector among the Z=100 vectors, thereby reducing the solution space *S* to 0.01%. With a streamlined solution space, it is feasible to calculate all *Z* vectors using the LS cost function, a more computationally complex method to further refine the estimation.

**Figure 8:**
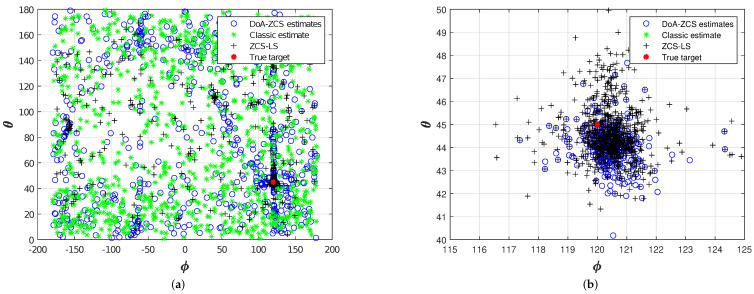
Comparison among Classic, ZCS, and ZCS-LS DoA estimations (1000 independent simulations).

Figure 8 depicts the DoA estimation results for 1000 trials. The accuracy rates obtained with simulated data are as follows: classic estimations = 0% [7], ZCS = 34%, and ZCS-LS = 74%. The classic estimation method fails to handle complex acoustic environments properly, i.e., when the primary peaks of the cross-correlations do not correspond to a specific source. This highlights the need for more sophisticated methods capable of accounting for secondary peaks that are complexities inherent to real-world acoustic environments.

### 6.3. DoA Estimation with Experimental Data

**Figure 9:**
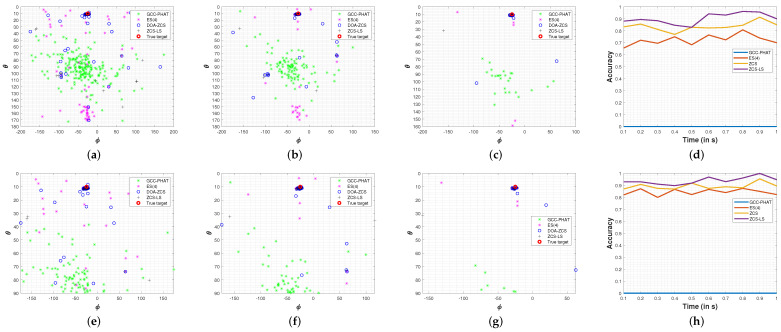
Experimental results: drone DoA estimates 100 m away from the microphone. (**a**) 209 estimations with 100 ms windows (ZCS-LS accuracy of 88.0%) and (**e**) 100 ms windows (ZCS-LS accuracy of 92.9% discarding estimations pointing to the floor); (**b**) 104 estimations with 200 ms windows (ZCS-LS accuracy of 89.4%) and (**f**) 200 ms windows (ZCS-LS accuracy of 93.0% discarding estimations pointing to the floor); (**c**) 104 estimations with 800 ms windows (ZCS-LS accuracy of 96.1%) and (**g**) 800 ms windows (ZCS-LS accuracy of 96.1% discarding estimations pointing to the floor); (**d**) accuracy of DoA estimators with different signal window sizes and (**h**) accuracy of DoA estimators with different signal window sizes (discarding estimations pointing to the floor).

Figure 9 illustrates the drone DoA estimation results using different window sizes. The GCC-PHAT method yielded 0% accuracy, indicating poor performance in handling the complexities of the acoustic environment. The ES(4) achieved an average accuracy of 81.1%, indicating that the exclusion of peaks that do not contribute to minimizing the error can significantly enhance the results. In contrast, the ZCS and ZCS-LS methods pointed toward the correct direction, achieving accuracies of =83.5%±3.6% for ZCS and =90.2%±4.4% for ZCS-LS. Using ZCS and ZCS-LS results in a high-density area of estimations around the actual angles, θ=10 and ϕ=−25. It should be noted that the ZCS facilitates exhaustive computations of all possible delay combinations, and the additional computation of the LS cost function enhances estimations by approximately 7%. The experimental outcomes surpassed the simulation’s, primarily attributable to the variable number of delay candidates (*C*) encountered. While the simulation phase maintained a fixed value of C=10, the experimental phase yielded a fluctuating range of peaks, ranging from 1 to 8 for each cross-correlation. This variability in delay candidates in the experimental setting contributed to the enhanced performance observed, demonstrating the method’s adaptability in real-world scenarios. The microphone array is positioned on the floor; thus, it is possible to exclude the wrong estimations pointing to the floor. This procedure enhances the utility of the method in real-world applications. Therefore, the experimental results of both ZCS and ZCS-LS techniques improved to 89.4%±2.7%, and 94.0%±3.1%, respectively.

## 7. Discussion

The main question addressed by our research is to unleash the potential of TDE-based DoA estimators by minimizing TDE errors using both primary and secondary peaks of the cross-correlation functions. Our previous work used genetic algorithms with a ZCS fitness function to perform a heuristic search for the best combination of peaks of the cross-correlations. Still, genetic algorithms often return a sub-optimal solution (this is the previously mentioned gap). In this paper, a method was devised to calculate the ZCS cost function of all combinations of delays to obtain the optimal solution related to this cost function. It was necessary to reduce the number of combinations of peaks of the cross-correlations by reducing the number of microphones in the array.

The proposed method tackles the problem by assuming that the correct delay between pairs of microphones can be the secondary peaks (peaks of the cross-correlations that do not have the greatest amplitude), which holds true, for instance, for highly noisy environments.

**First stage:** comprises the usage of a low complexity ZCS cost function to perform an exhaustive search (this low complexity cost function is adequate for exhaustive searches because it primarily involves matrix multiplication), i.e., tests all combinations of ${\hat{\tau }}_{ij}$ (estimated delays) with the ZCS cost function from all peaks of each cross-correlation, ${\hat{r}}_{x_{i}x_{j}}$. This is how we leverage secondary peaks, by taking them into account while performing the exhaustive search. **Secon dstage:** uses a least squares cost function only in a subset of the combination of delays that hold the lowest ZCS cost function. By doing this, the computational complexity is reduced and the accuracy of the DoA estimation is enhanced. The ZCS condition was used to implement a reduced complexity cost function to perform a first evaluation of all combinations of delays. In the absence of noise, i.e., when the theoretical delays $\tau_{ij}$ are available, the DoA estimations could be achieved flawlessly using the ZCS.

The LS solution refines the DoA estimation process by selecting one combination of the delays that has the lowest ZCS cost function and minimizes the LS cost function. We examined the first delay combinations with the lowest ZCS and observed that the best combination of delays was among the 100 lowest ZCS cost functions for the drone signals database. The idea of using the LS cost function was raised from this observation. With this cost function, it is possible to evaluate each combination (within the reduced set of candidates that has the lowest ZCS) through the mean squared error of the difference between the theoretical delays and the estimated delays. This cost function is more complex and demands more computational efforts, thus it is suitable only for a very small set of delay combinations.

Experiments were conducted using simulated signals and experimental data. In both experiments, the performance of the proposed method was evaluated by estimating the DoA of the drone. Estimations were considered correct if both the error in the estimated zenith ($\hat{\theta }$) and estimated azimuth ($\hat{\phi }$) were within $\pm 5^{\circ }$.

The proposed method yields this accuracy because it considers the secondary peaks as candidates of true delay estimations and uses this information to perform DoA estimations using the above-mentioned cost functions in a two-stage method. Conversely, the GCC-PHAT method does not take into consideration secondary peaks. According to our investigations, complex acoustic scenarios tend to yield cross-correlation functions in which the estimated delay is one of the many secondary peaks of each cross-correlation.

The parameter of the proposed method that yields this accuracy is the secondary peaks of each cross-correlation, i.e., each plausible solution to the DoA estimation problem is represented as a column within matrix $V_{N\times C}$. The GCC-PHAT method only tests the primary peaks, i.e., the first column of matrix $V_{N\times C}$.

The lowest ZCS cost function alone does not guarantee the best accuracy in terms of DoA. We found in our experiments that the best set of delays was among the 100 lowest ZCS scores. This discovery prompted us to introduce a second stage employing the LS cost function to enhance the results, resulting in significant improvements.

The ZCS and LS solutions often align and represent optimal solutions. However, sometimes the ZCS solution may represent a sub-optimal solution. In such cases, we discovered that the LS could be utilized to select an enhanced set of delays improving the results, i.e., an additional computational effort can benefit the DoA estimation.

## 8. Conclusions

In this work, we focused on acoustic source DoA estimation, i.e., the challenging backdrop of heavily noisy environments. This investigation focused on developing and validating a DoA estimation method based on finding the correct delays estimated using the cross-correlation function. The simulation results showed that the simulated data represented complex environments with many problems that could invalidate the DoA estimation. However, an exhaustive search with a low complex cost function diminished the vast solution space and raised the results by 34 percentage points. The second stage of the method enhanced the results by 74 percentage points. This method is, thus, applicable to heavily noisy signals whenever the time delay information is present in the first ten peaks. The experimental findings unveiled the applicability of the ZCS and ZCS-LS methods to actual signals with accuracies of 83.5%±3.6% and 90.2%±4.4%, respectively. Given that the microphone array is positioned on the floor, it becomes feasible to eliminate numerous estimations pointing toward the floor, indicated by $\theta >90^{\circ }$.

Consequently, the results obtained with these methods in a real-world scenario are anticipated to exhibit a notable increase in accuracy, benefiting from discarding estimations that do not align with the physical orientation of the microphone array. By implementing this refinement, a singular high-density area of estimations persists. The selective exclusion of estimations pointing toward the floor ensures a focused and accurate representation of potential drone locations. This precision enhances the practical utility of the methods in real-world applications. Following this refinement step, the experimental results exhibit improvement, with ZCS accuracy reaching 89.4%±2.7% and ZCS-LS accuracy reaching 94.0%±3.1%.

## Figures and Tables

**Table 1:** All possible cyclic paths in a four-microphone array.

# Delays	Closed Loops			
3	$\tau_{12}$	$\tau_{23}$	$\tau_{31}$	
	$\tau_{12}$	$\tau_{24}$	$\tau_{41}$	
	$\tau_{13}$	$\tau_{34}$	$\tau_{41}$	
	$\tau_{23}$	$\tau_{34}$	$\tau_{42}$	
4	$\tau_{12}$	$\tau_{23}$	$\tau_{34}$	$\tau_{41}$

## Data Availability

No new data were created or analyzed in this study. Data sharing is not applicable to this article.
